# Supplementary material for: A Novel Microencapsulated Bovine Recombinant Interferon Tau Formulation for Luteolysis Modulation in Cattle
Source: Biomolecules. 2025 Jul 14;15(7):1009. doi: 10.3390/biom15071009 (PMC12293041; doi:10.3390/biom15071009)
Supplement: Supplementary file 1 [file biomolecules-15-01009-s001.zip › File S1. Western Blotting Figures..pdf]

Supplementary materials

Figure 3A. SDS-PAGE and *Western blot*.

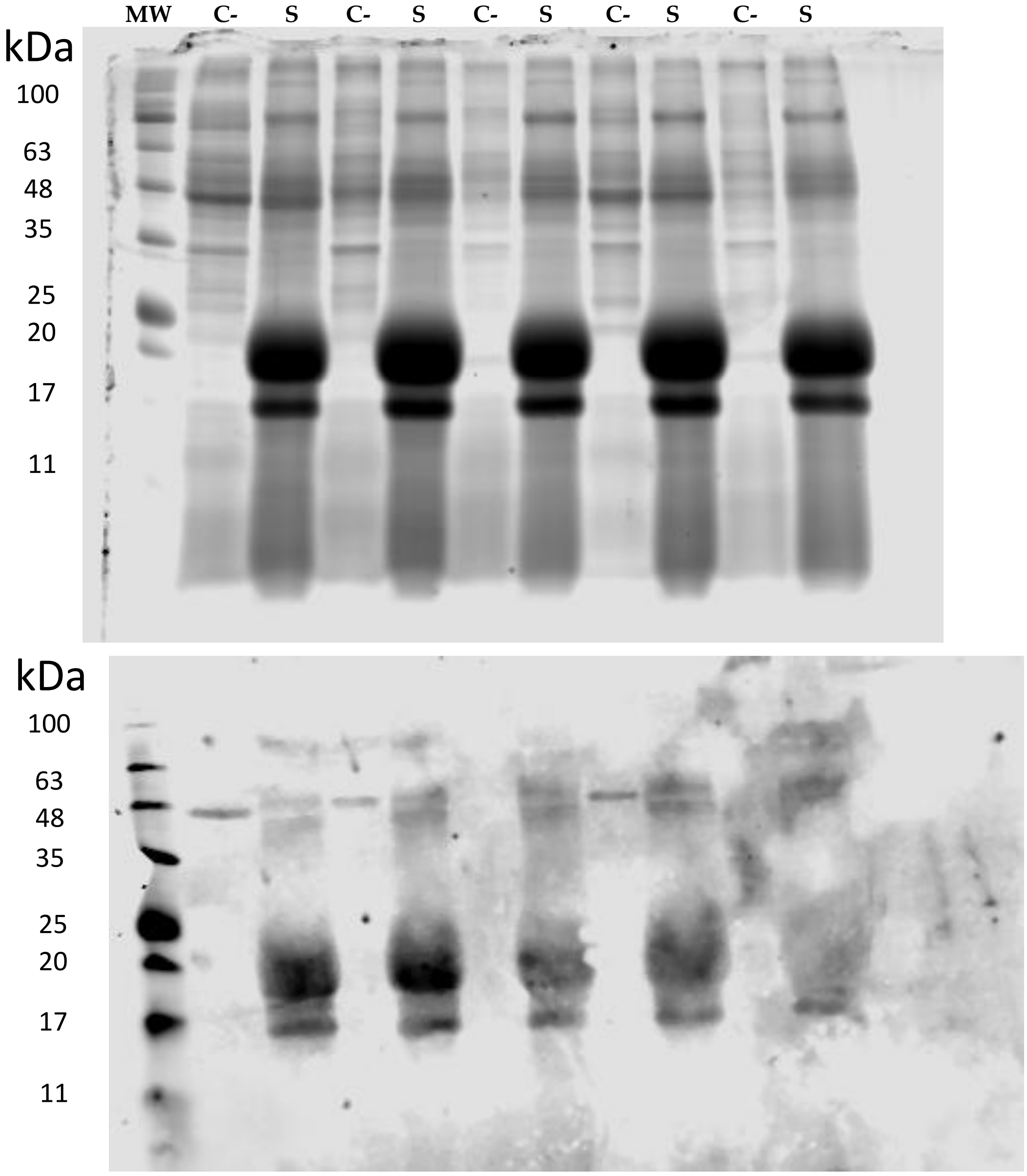

Figure 3B. SDS-PAGE.

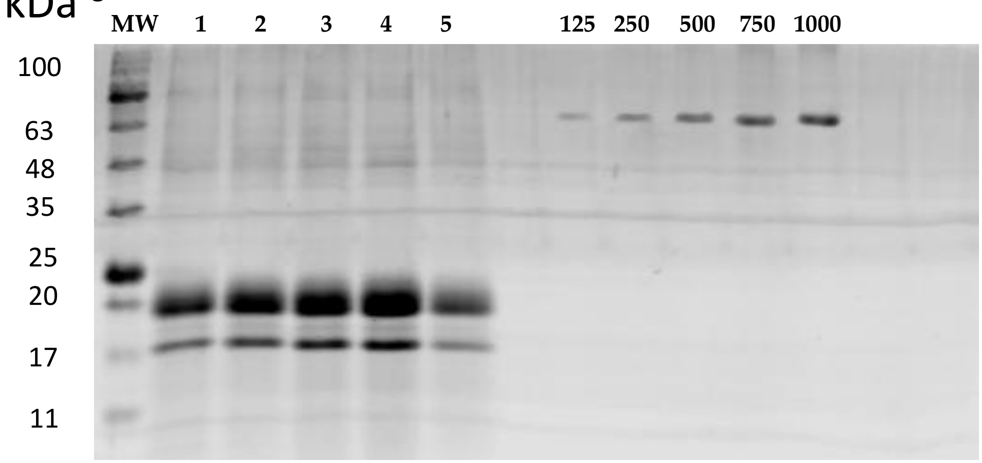

Figure 3C. SDS-PAGE and *Western blot.*

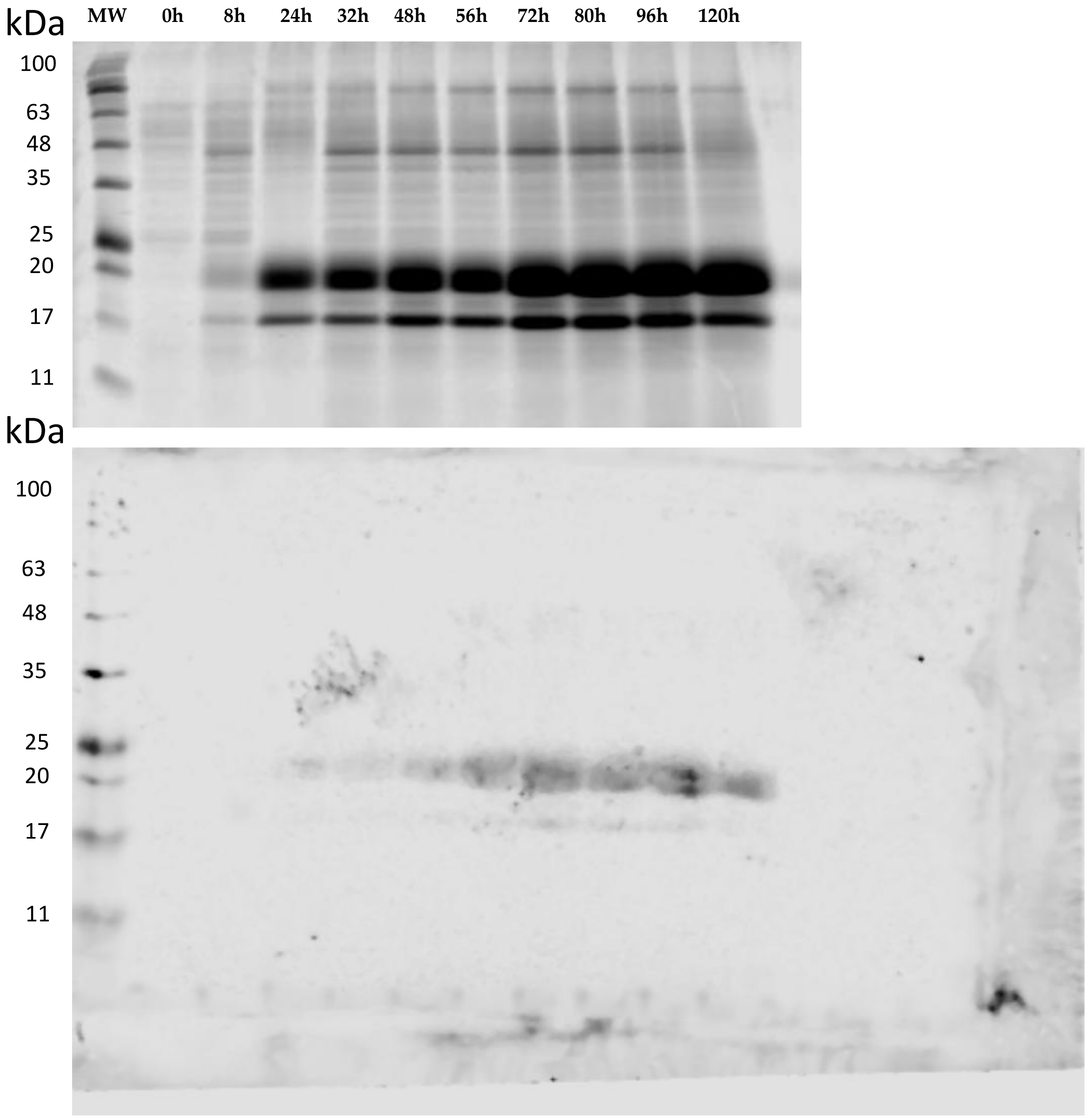

Figure 3D. SDS-PAGE and *Western blot.*

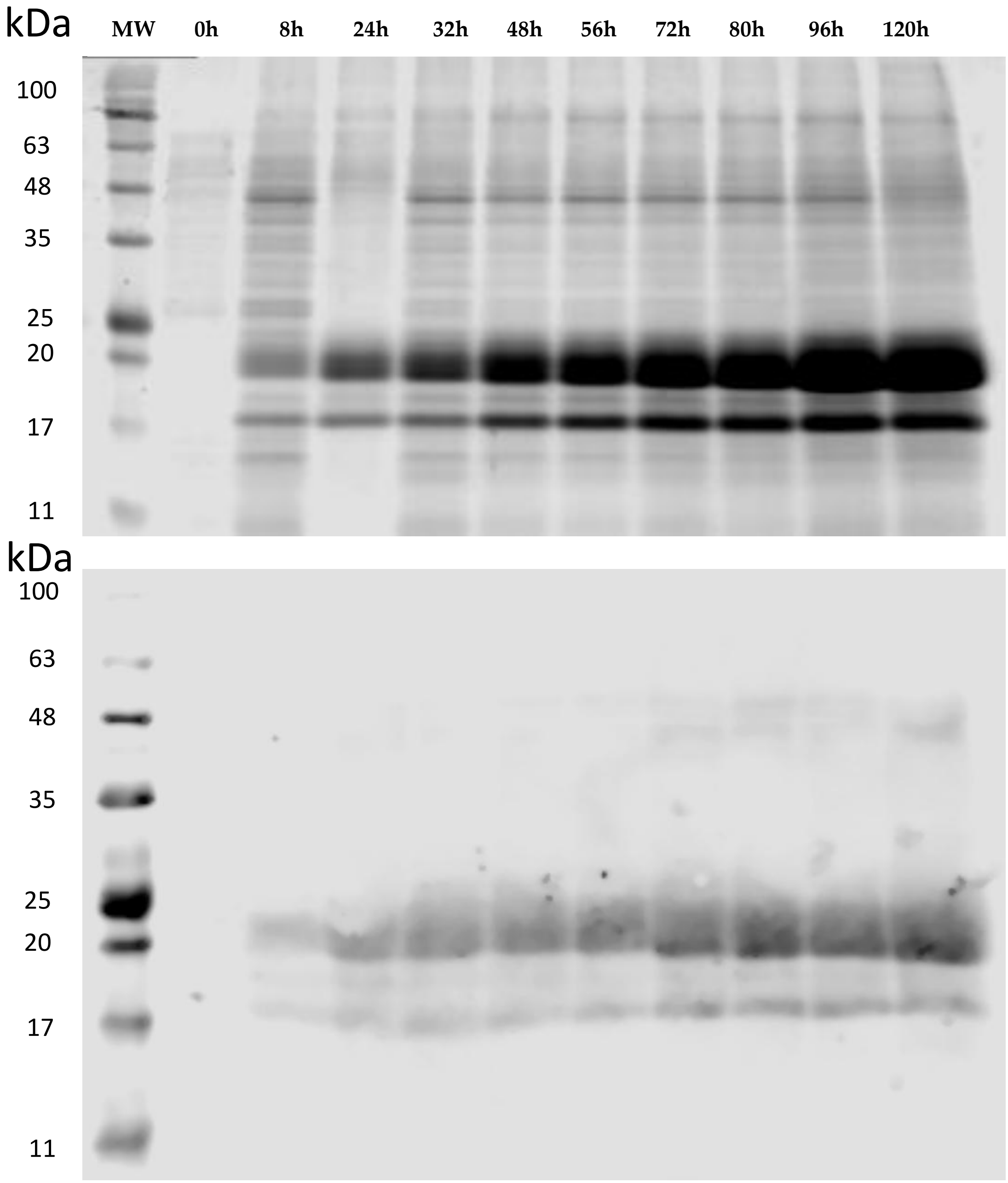

Figure 4C. SDS-PAGE and *Western blot*

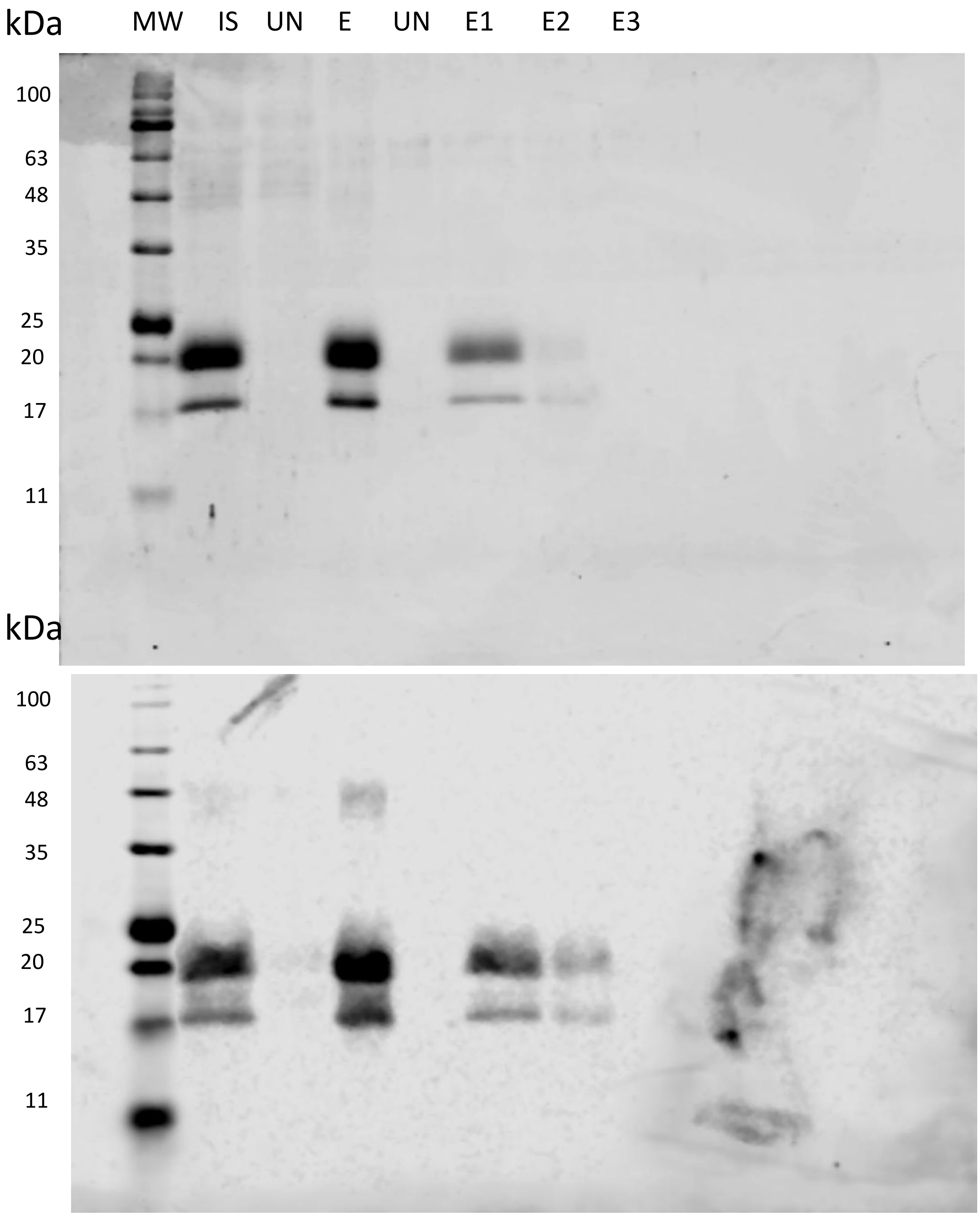

Figure 6H. *Western blot*.

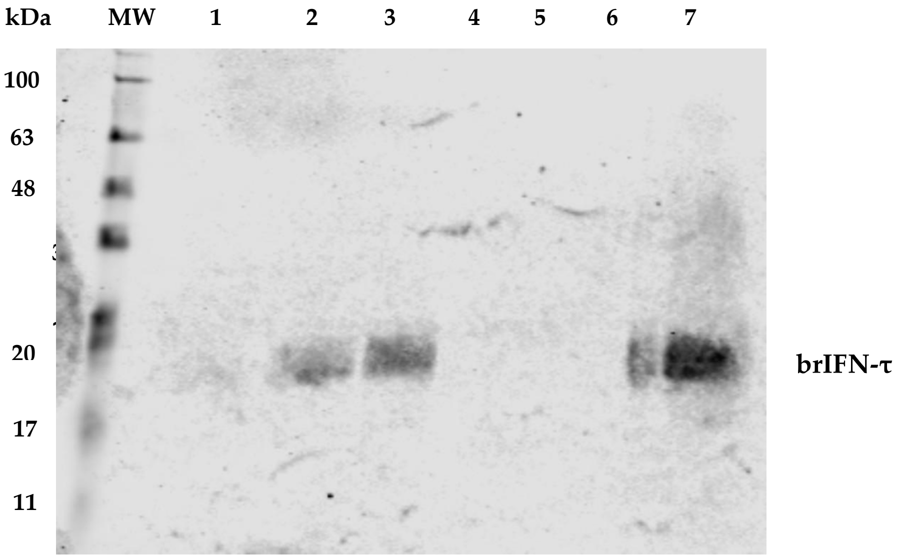

Figure 7B. *Western blot*

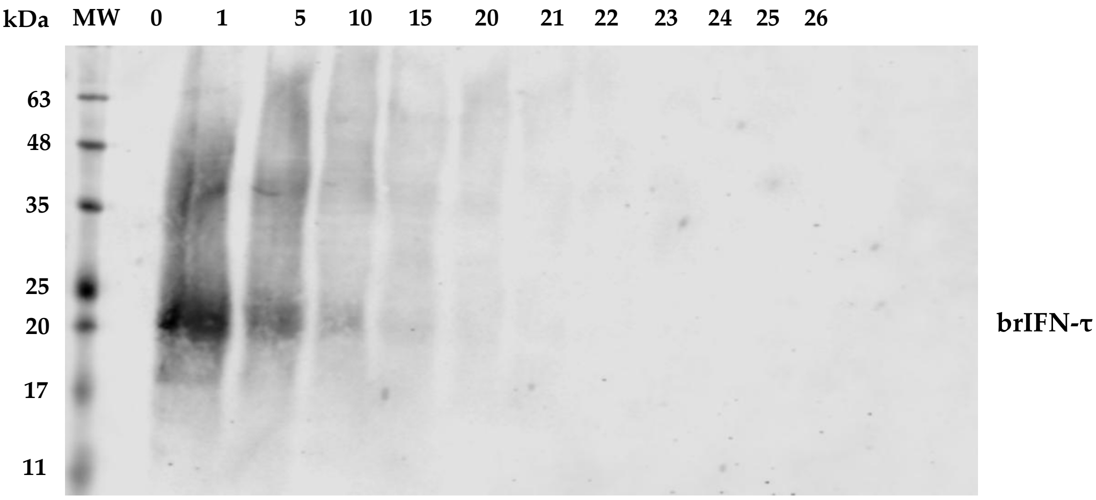

Image ID 0029750_05
14 de mayo de 2024

Image Display Parameters

Channel	Color	Minimum	Maximum	K
700	Gray Scale (Black on White)	67,0	617	0,13

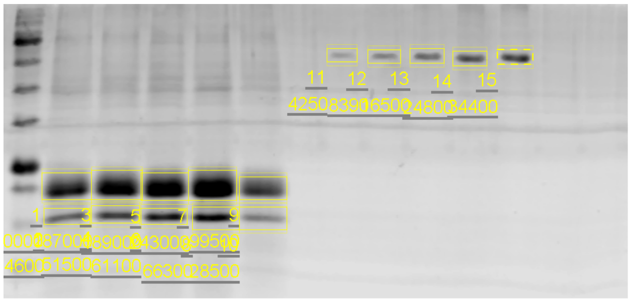

Acquisition Information

Column	Value
Image ID	0029750_05
Acquire Time	Tue May 14 17:07:58 CLT 2024
Channels	700 800
Resolution	169um
Intensities	4 4
Quality	medium
Analysis	Manual
Image Name	0029750_01
Comment	14052024 SDS IFN TAU C1 C2 C3 C4 C5
Image Modifications	Noise Removal Image ID: 0029750_01; Rotate 180 Image ID: 0029750_02; Crop Image ID: 0029750_03; Crop Image ID: 0029750_04

All Shapes

#	Image Name	Channel	Name	Signal	Total	Area	Bkgnd.	Type	Conc. Std.	Concentration
1	0029750_01	700	00001	140000	257000	760	153	Signal	NaN	NaN
2	0029750_01	700	00002	34600	91600	420	136	Signal	NaN	NaN
3	0029750_01	700	00003	187000	321000	858	155	Signal	NaN	NaN
4	0029750_01	700	00004	51500	119000	481	140	Signal	NaN	NaN
5	0029750_01	700	00005	189000	330000	756	187	Signal	NaN	NaN
6	0029750_01	700	00006	61100	125000	420	152	Signal	NaN	NaN
7	0029750_01	700	00007	243000	399000	851	184	Signal	NaN	NaN
8	0029750_01	700	00008	66300	164000	612	159	Signal	NaN	NaN
9	0029750_01	700	00009	99500	196000	612	158	Signal	NaN	NaN
10	0029750_01	700	00010	28500	101000	612	118	Signal	NaN	NaN
11	0029750_01	700	00011	4250	37100	230	143	Signal	NaN	NaN
12	0029750_01	700	00012	8390	46100	250	151	Signal	NaN	NaN
13	0029750_01	700	00013	16500	58700	286	148	Signal	NaN	NaN
14	0029750_01	700	00014	24800	68600	286	153	Signal	NaN	NaN
15	0029750_01	700	00015	34400	76200	297	141	Signal	NaN	NaN

Image ID 0030079_06
12 de junio de 2024

Image Display Parameters

Channel	Color	Minimum	Maximum	K
700	Gray Scale (Black on White)	31,9	570	0,48

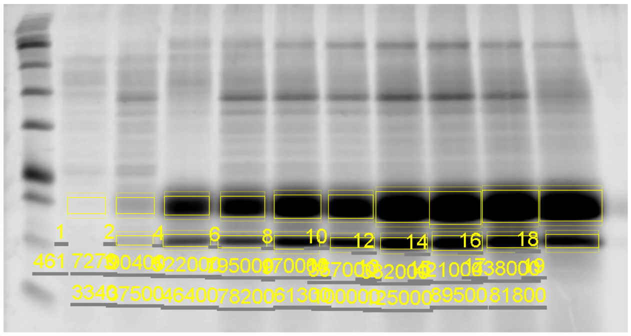

Acquisition Information

Column	Value
Image ID	0030079_06
Acquire Time	Fri Jun 07 11:28:03 CLT 2024
Channels	700 800
Resolution	169um
Intensities	4 4
Quality	medium
Analysis	Manual
Image Name	0030079_01
Comment	04062024 CURVA DE EXPRESION C3
Image Modifications	Rotate 180 Image ID: 0030079_01; Crop Image ID: 0030079_02; Noise Removal Image ID: 0030079_03; Crop Image ID: 0030079_04; Crop Image ID: 0030079_05

All Shapes

#	Image Name	Channel	Name	Signal	Total	Area	Bkgnd.	Type	Conc. Std.	Concentration
1	0030079_01	700	00001	461	11000	286	37,0	Signal	NaN	NaN
2	0030079_01	700	00002	7270	29400	286	77,4	Signal	NaN	NaN
3	0030079_01	700	00003	3340	12600	150	61,6	Signal	NaN	NaN
4	0030079_01	700	00004	90400	178000	390	225	Signal	NaN	NaN
5	0030079_01	700	00005	37500	62600	256	98,3	Signal	NaN	NaN
6	0030079_01	700	00006	122000	217000	420	227	Signal	NaN	NaN
7	0030079_01	700	00007	46400	75000	264	108	Signal	NaN	NaN
8	0030079_01	700	00008	195000	323000	512	250	Signal	NaN	NaN
9	0030079_01	700	00009	78200	116000	279	134	Signal	NaN	NaN
10	0030079_01	700	00010	170000	282000	480	232	Signal	NaN	NaN
11	0030079_01	700	00011	61300	93800	210	155	Signal	NaN	NaN
12	0030079_01	700	00012	357000	550000	756	255	Signal	NaN	NaN
13	0030079_01	700	00013	100000	158000	256	225	Signal	NaN	NaN
14	0030079_01	700	00014	382000	602000	770	285	Signal	NaN	NaN
15	0030079_01	700	00015	125000	192000	340	197	Signal	NaN	NaN
16	0030079_01	700	00016	421000	653000	836	278	Signal	NaN	NaN
17	0030079_01	700	00017	89500	160000	272	260	Signal	NaN	NaN
18	0030079_01	700	00018	438000	663000	882	255	Signal	NaN	NaN
19	0030079_01	700	00019	81800	145000	324	195	Signal	NaN	NaN

LI-COR
Experience Excellence

Image ID 0030077_09
12 de junio de 2024

Image Display Parameters

Channel	Color	Minimum	Maximum	K
700	Gray Scale (Black on White)	40,8	845	0,41

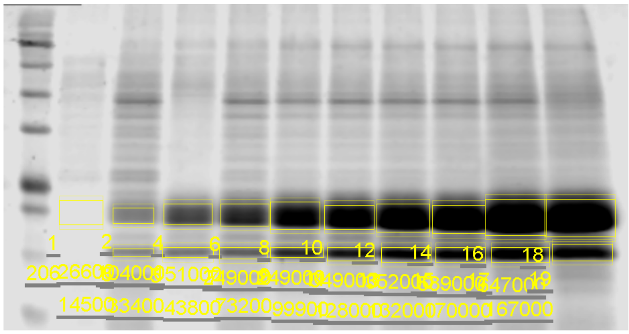

Acquisition Information

Column	Value
Image ID	0030077_09
Acquire Time	Fri Jun 07 11:03:22 CLT 2024
Channels	700 800
Resolution	169um
Intensities	4 4
Quality	medium
Analysis	Manual
Image Name	0030077_01
Comment	04062024 CURVA DE EXPRESION C4 (*c2*)
Image Modifications	Crop Image ID: 0030077_01; Noise Removal Image ID: 0030077_02; Flip Top to Bottom Image ID: 0030077_03; Crop Image ID: 0030077_04; Crop Image ID: 0030077_05; Free Rotate 359 Image ID: 0030077_06; Crop Image ID: 0030077_07; Crop Image ID: 0030077_08

All Shapes

#	Image Name	Channel	Name	Signal	Total	Area	Bkgnd.	Type	Conc. Std.	Concentration
1	0030077_01	700	00001	206	17200	464	36,6	Signal	NaN	NaN
2	0030077_01	700	00002	26600	71000	270	164	Signal	NaN	NaN
3	0030077_01	700	00003	14500	30700	135	120	Signal	NaN	NaN
4	0030077_01	700	00004	104000	186000	448	183	Signal	NaN	NaN
5	0030077_01	700	00005	33400	55300	238	92,3	Signal	NaN	NaN
6	0030077_01	700	00006	151000	254000	480	215	Signal	NaN	NaN
7	0030077_01	700	00007	43800	73900	256	118	Signal	NaN	NaN
8	0030077_01	700	00008	249000	393000	594	243	Signal	NaN	NaN
9	0030077_01	700	00009	73200	116000	245	175	Signal	NaN	NaN
10	0030077_01	700	00010	249000	419000	528	323	Signal	NaN	NaN
11	0030077_01	700	00011	99900	144000	279	159	Signal	NaN	NaN
12	0030077_01	700	00012	349000	571000	665	334	Signal	NaN	NaN
13	0030077_01	700	00013	128000	195000	330	204	Signal	NaN	NaN
14	0030077_01	700	00014	352000	609000	665	385	Signal	NaN	NaN
15	0030077_01	700	00015	132000	208000	330	230	Signal	NaN	NaN
16	0030077_01	700	00016	589000	988000	984	405	Signal	NaN	NaN
17	0030077_01	700	00017	170000	278000	390	278	Signal	NaN	NaN
18	0030077_01	700	00018	647000	1100000	1175	389	Signal	NaN	NaN
19	0030077_01	700	00019	167000	288000	440	276	Signal	NaN	NaN

Image ID: 0029918_09
Acquire Time: 26 may. 2024 16:46:34

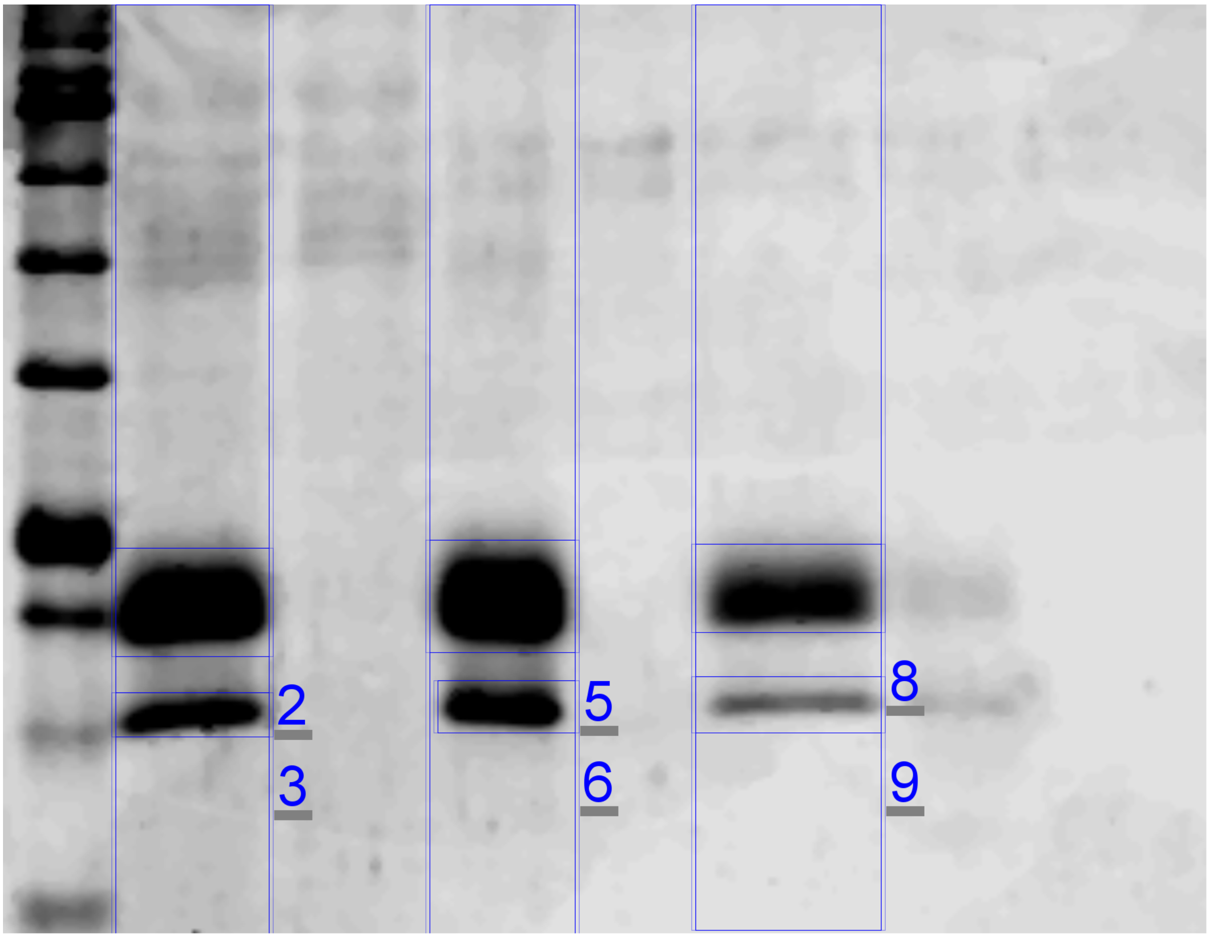

All Shapes

#	Image Name	Channel	Name	Signal	Total	Area	Bkgnd.	Type
1	0029918_01	700	00004	303000	627000	8316	39,0	Signal
2	0029918_01	700	00001	284000	723000	8778	50,0	Signal
3	0029918_01	700	00005	188000	244000	1008	56,0	Signal
4	0029918_01	700	00002	153000	241000	1026	86,0	Signal
5	0029918_01	700	00007	111000	502000	10580	37,0	Signal
6	0029918_01	700	00008	83900	131000	1012	47,0	Signal
7	0029918_01	700	00006	56700	76600	442	45,0	Signal
8	0029918_01	700	00003	37900	70500	418	78,0	Signal
9	0029918_01	700	00009	16400	42800	644	41,0	Signal

Image Information

Column	Value
Image ID	0029918_09
Acquire Time	26 may. 2024 16:46:34
Channels	700, 800
Resolution	169um
Intensities	4 4
Image Name	0029918_01

Image Information (continued)

Column	Value
Comment	26052024 SDS PURIFICACION IFN IC IA 3.1
Image Modifications	Noise Removal Image ID: 0029918_01; Crop Image ID: 0029918_02; Rotate 180 Image ID: 0029918_03; Noise Removal Image ID: 0029918_04; Crop Image ID: 0029918_06; Crop Image ID: 0029918_07; Crop Image ID: 0029918_08
